# Supplementary figures and images for: Visual landmarks sharpen grid cell metric and confer context specificity to neurons of the medial entorhinal cortex
Source: eLife. 2016 Jul 23;5:e16937. doi: 10.7554/eLife.16937 (PMC4987135; doi:10.7554/eLife.16937)

## Slide 1
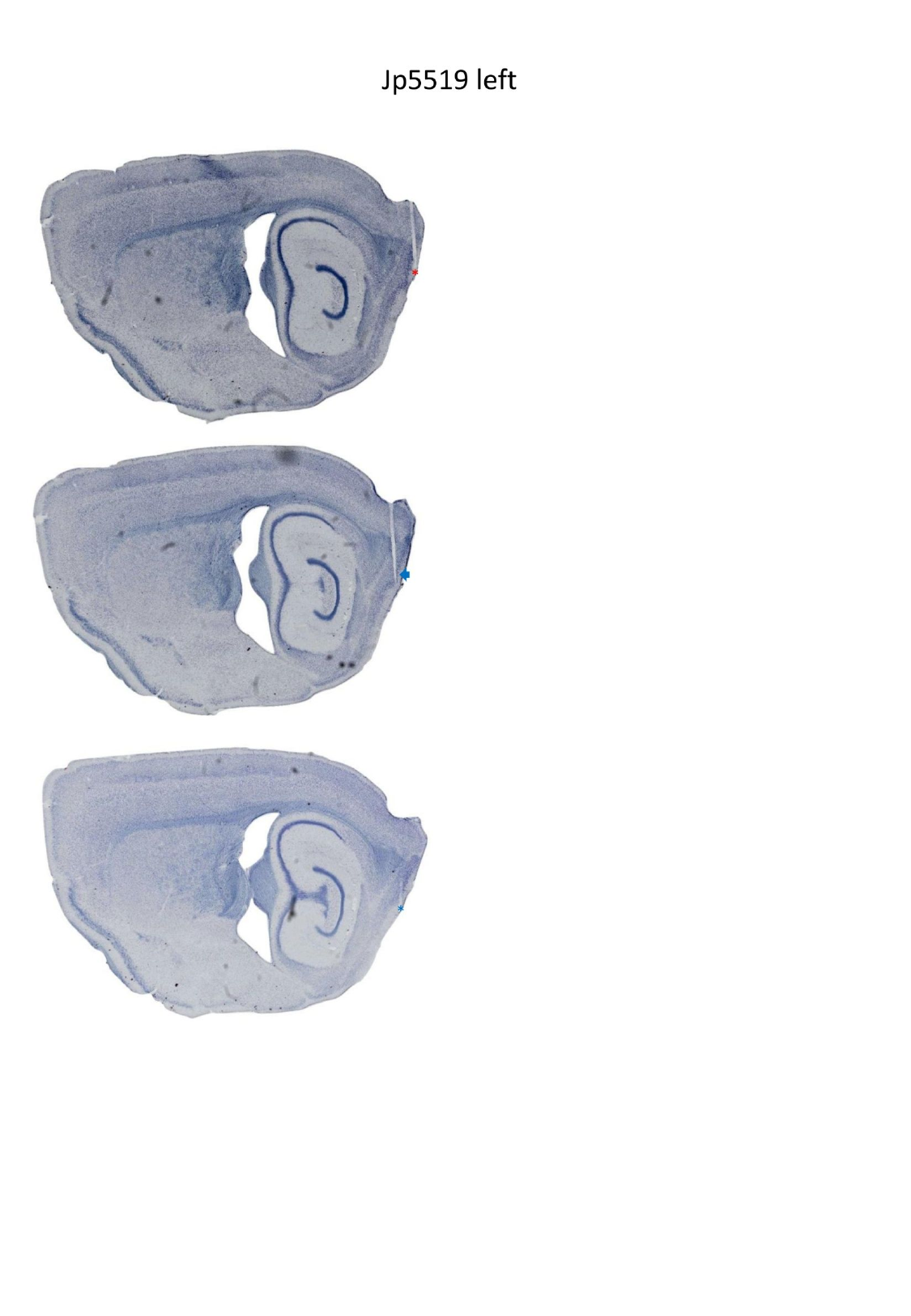

## Slide 2
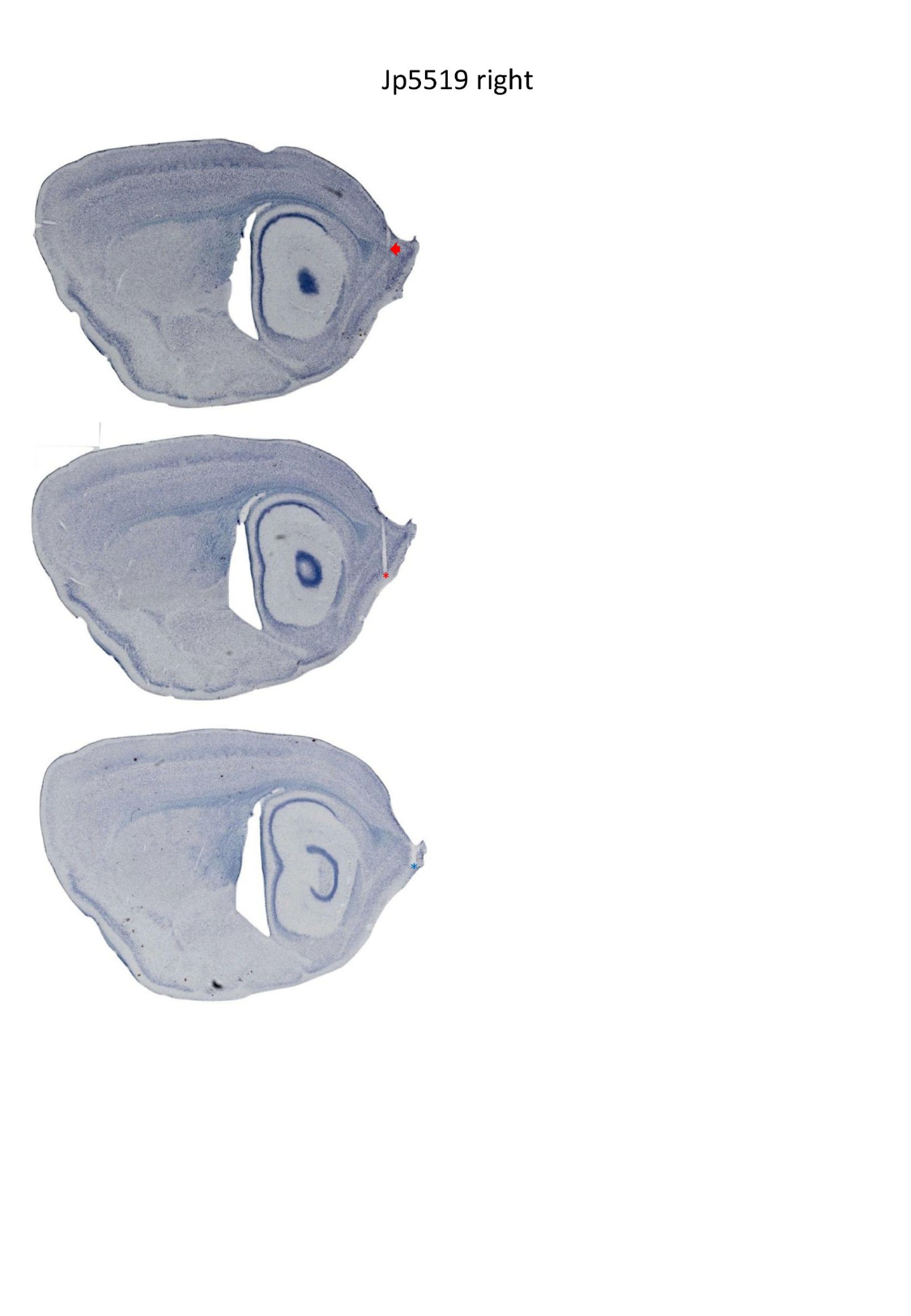

## Slide 3
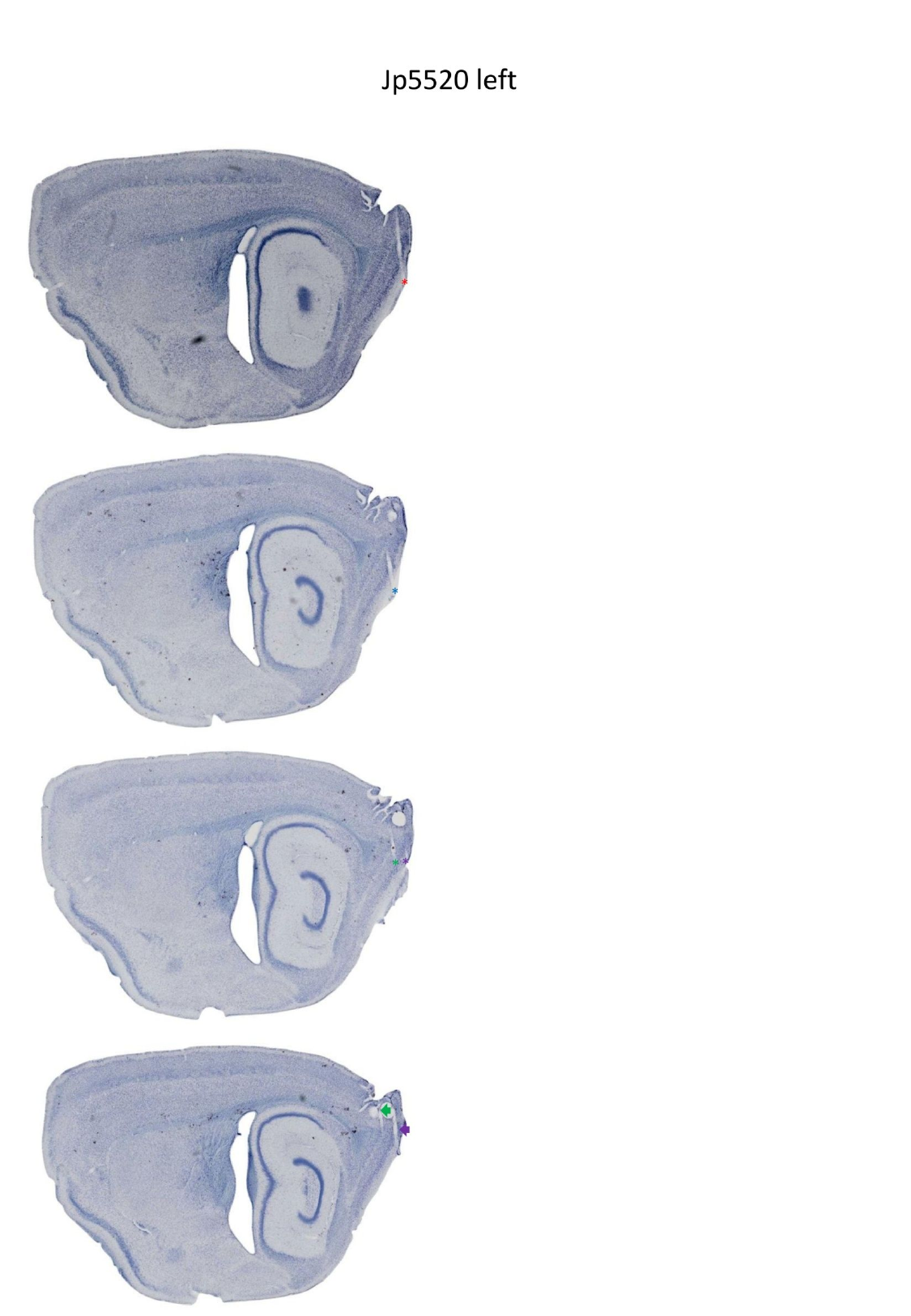

## Slide 4
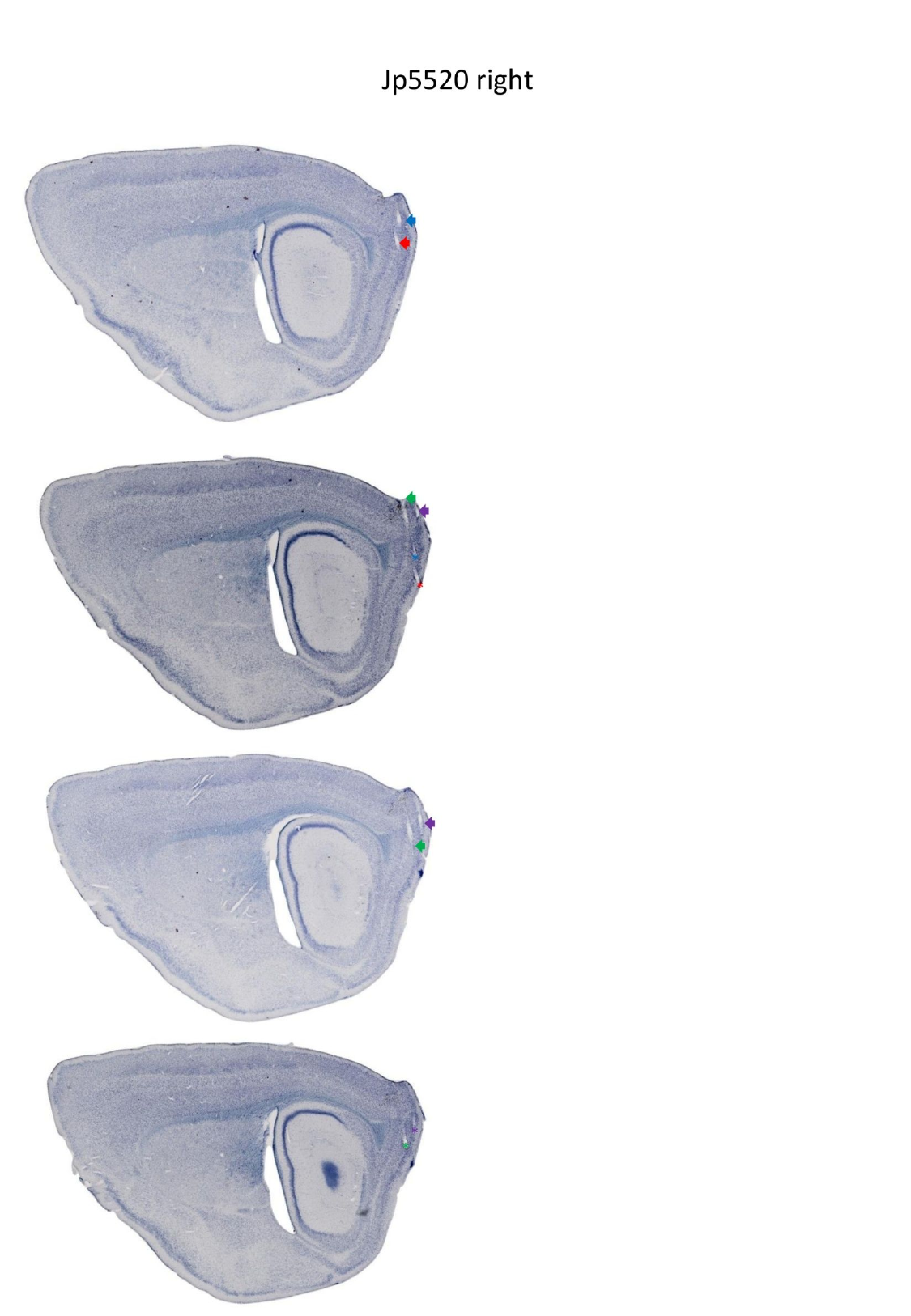

## Slide 5
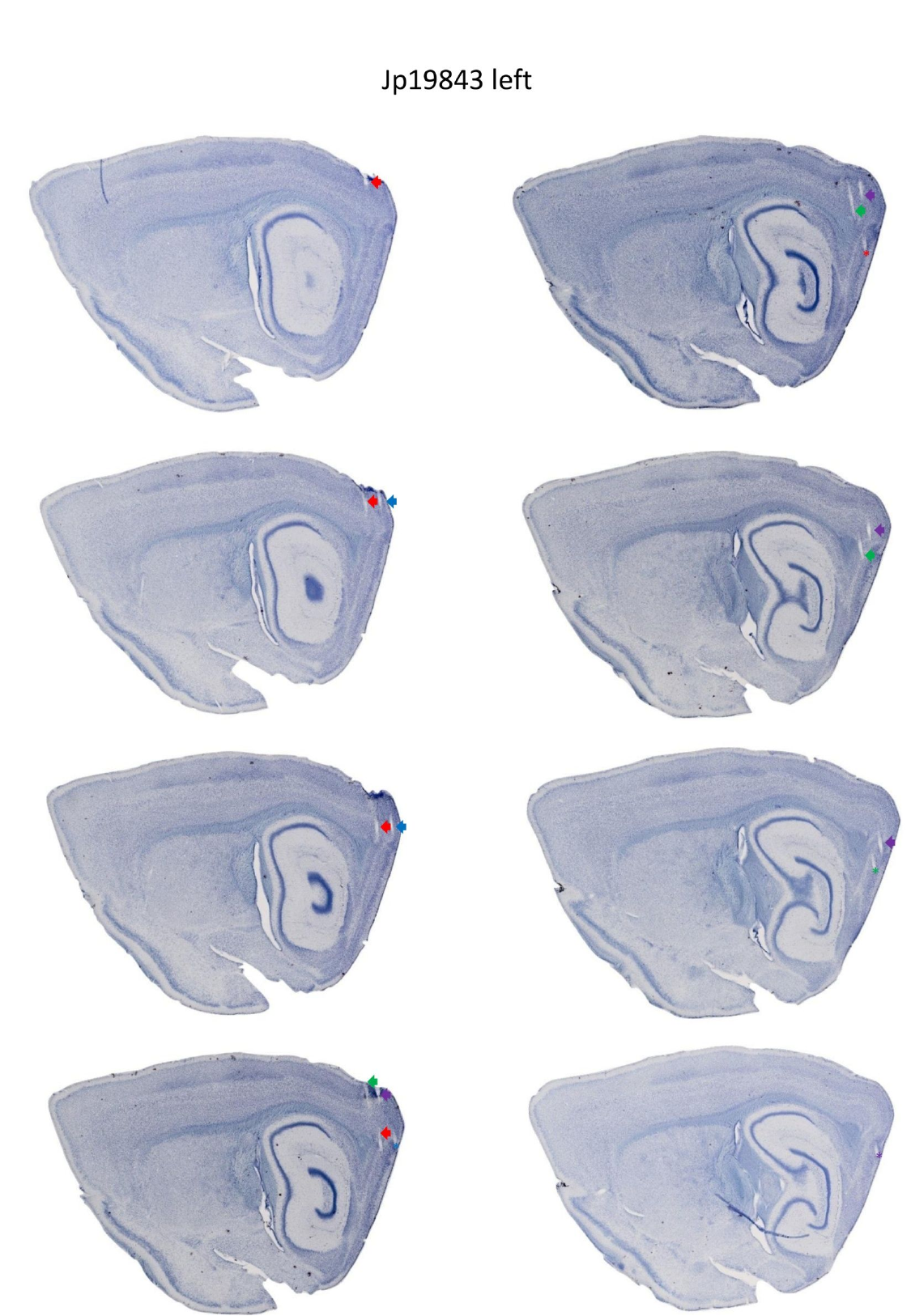

## Slide 6
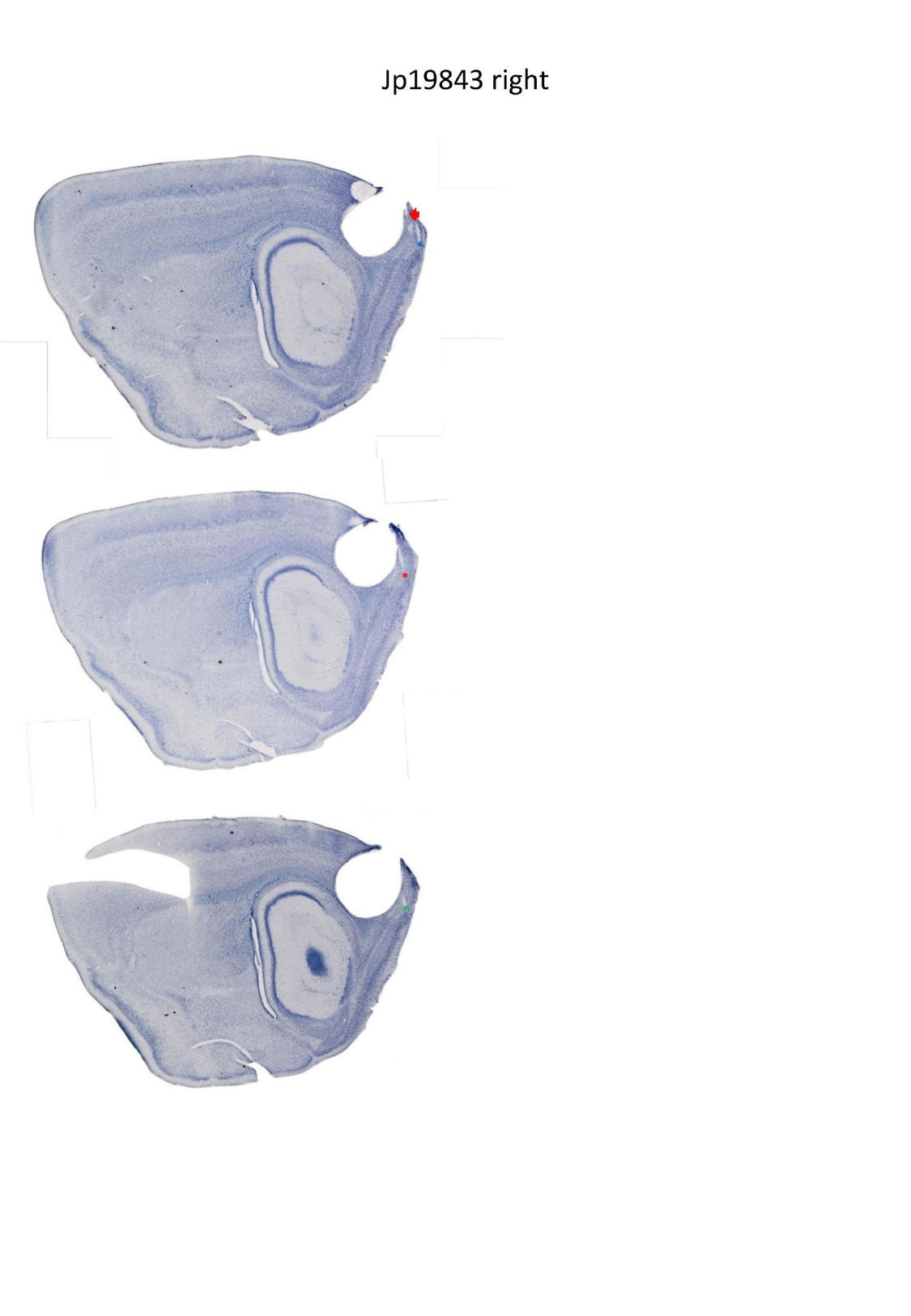

## Slide 7
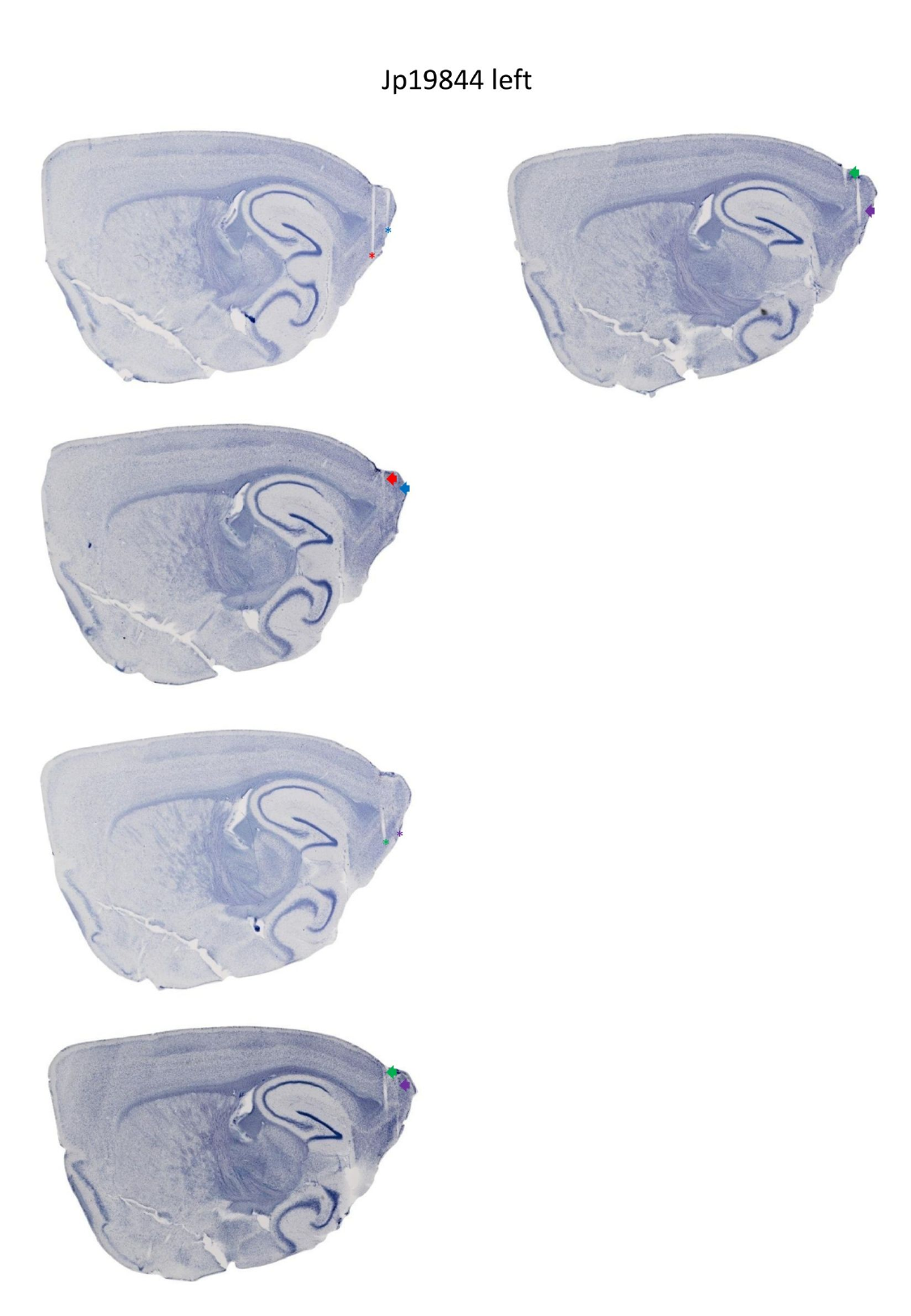

## Slide 8
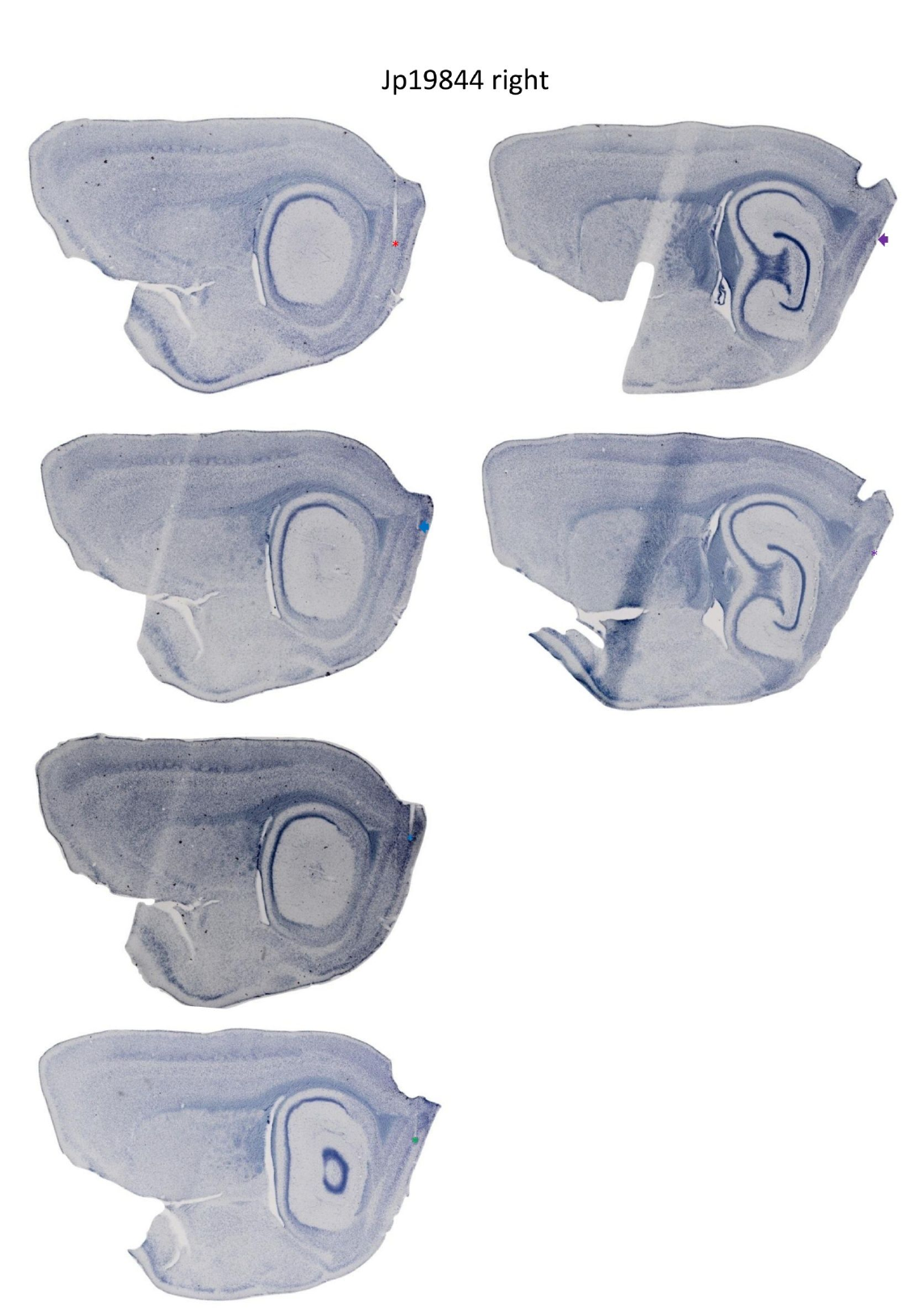

## Slide 9
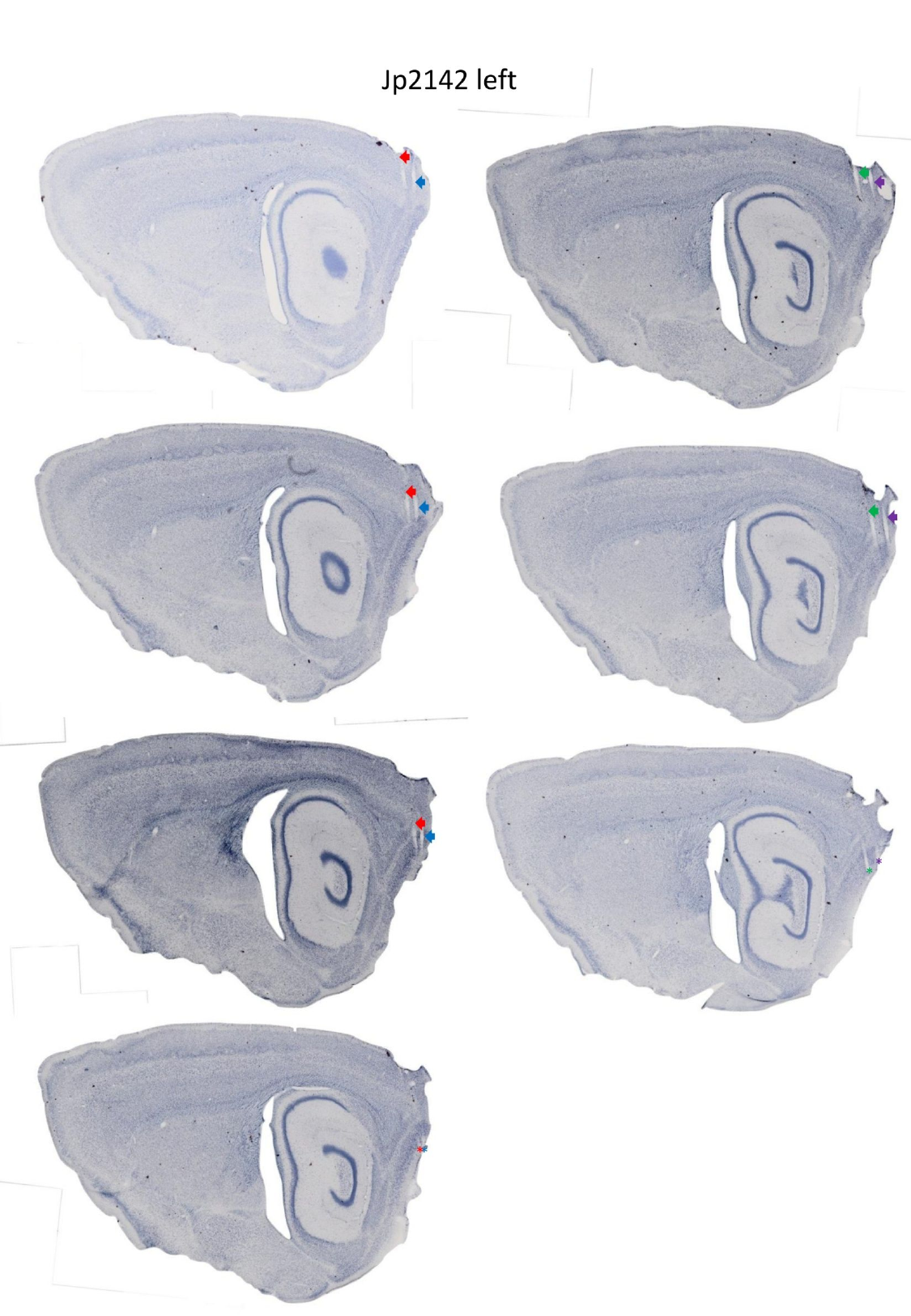

## Slide 10
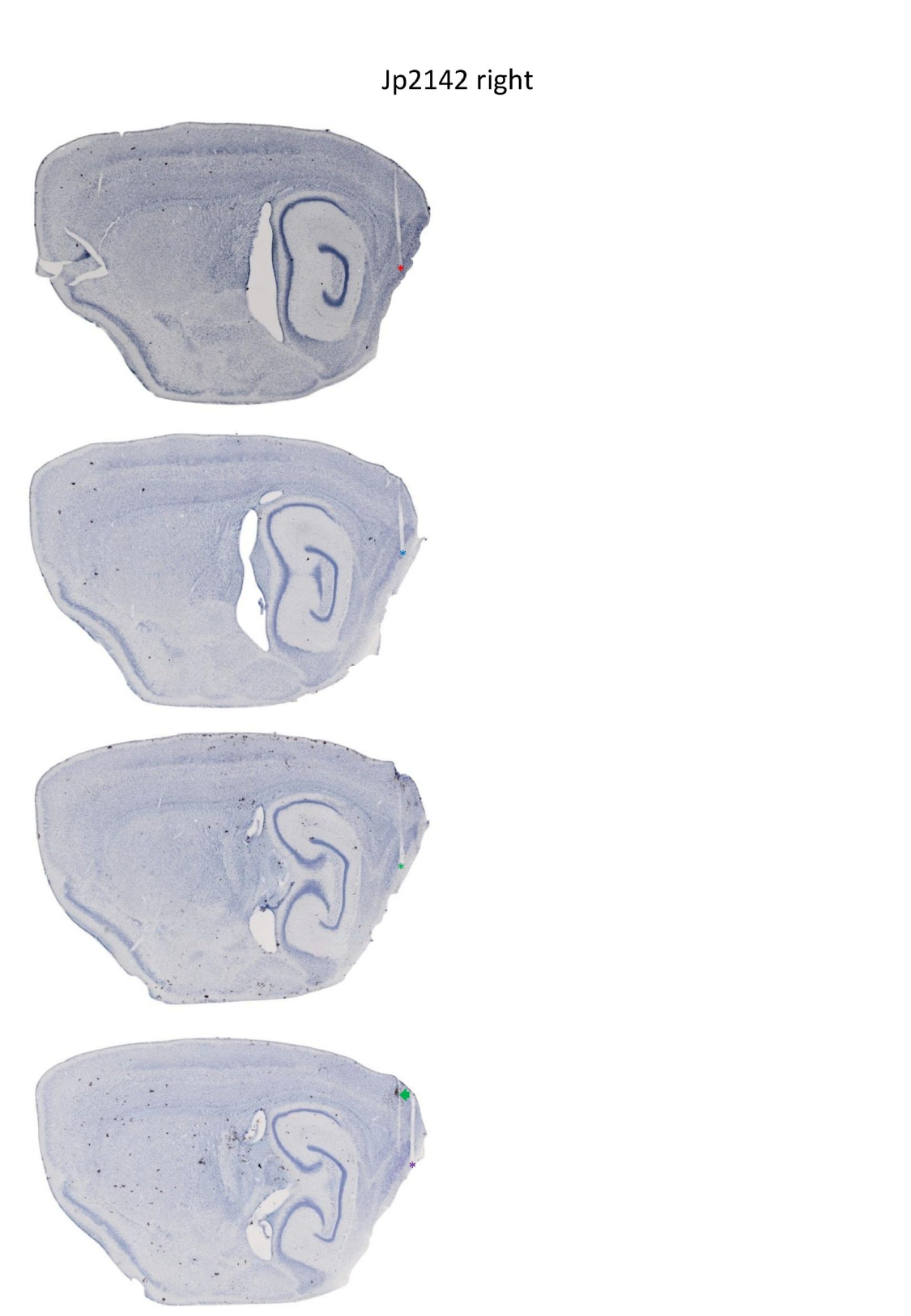

## Slide 11
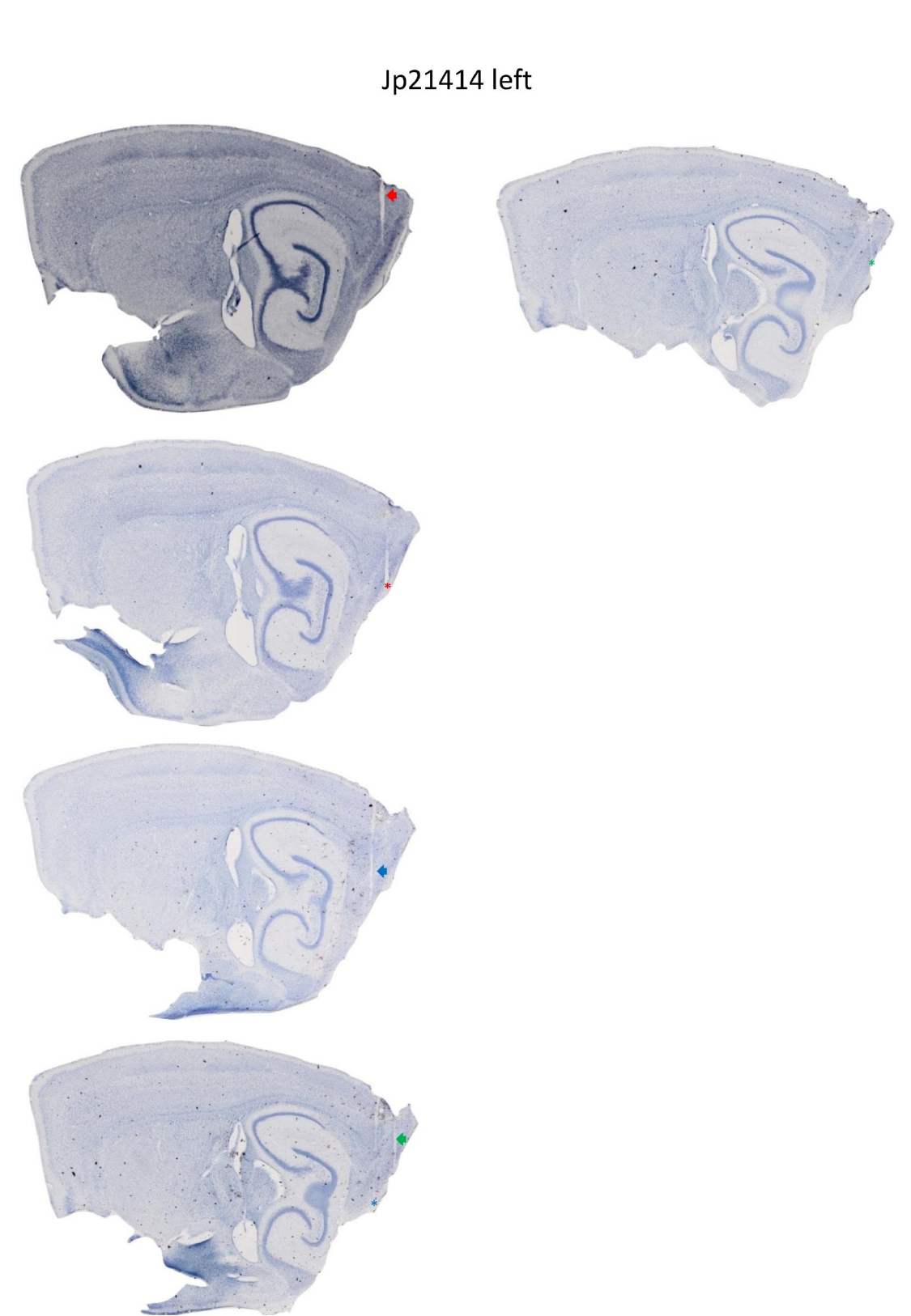

## Slide 12
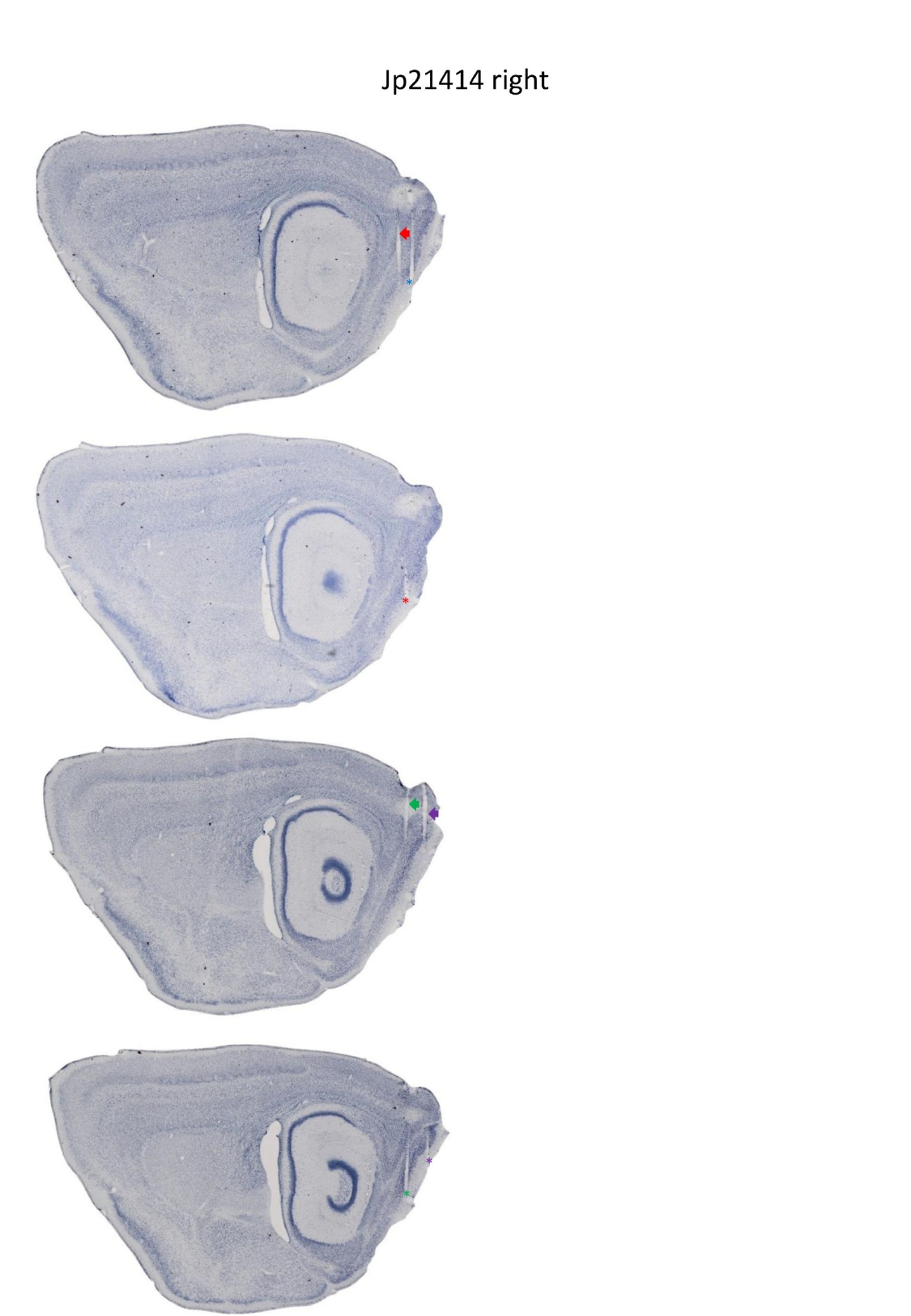

## Slide 13
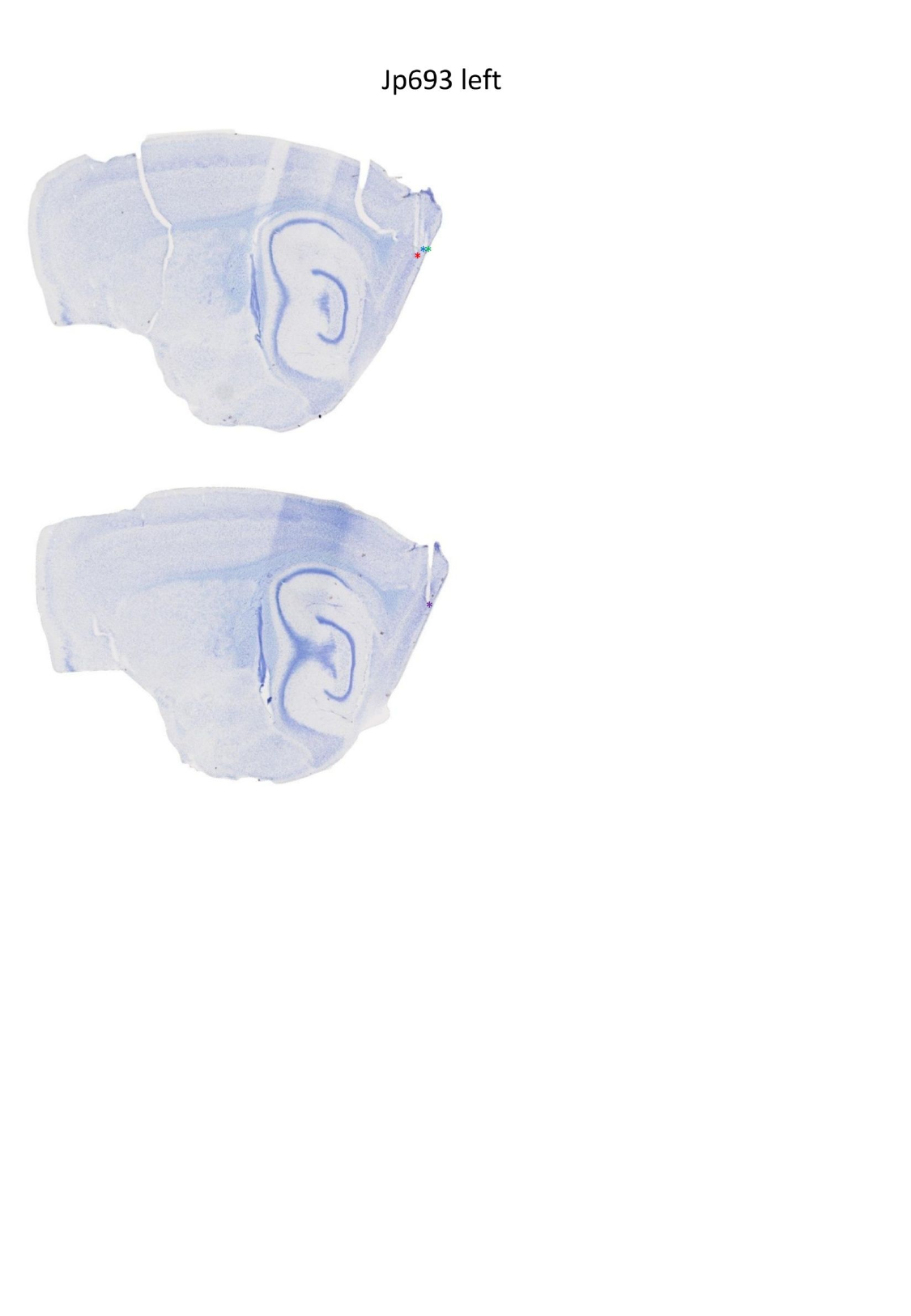

## Slide 14
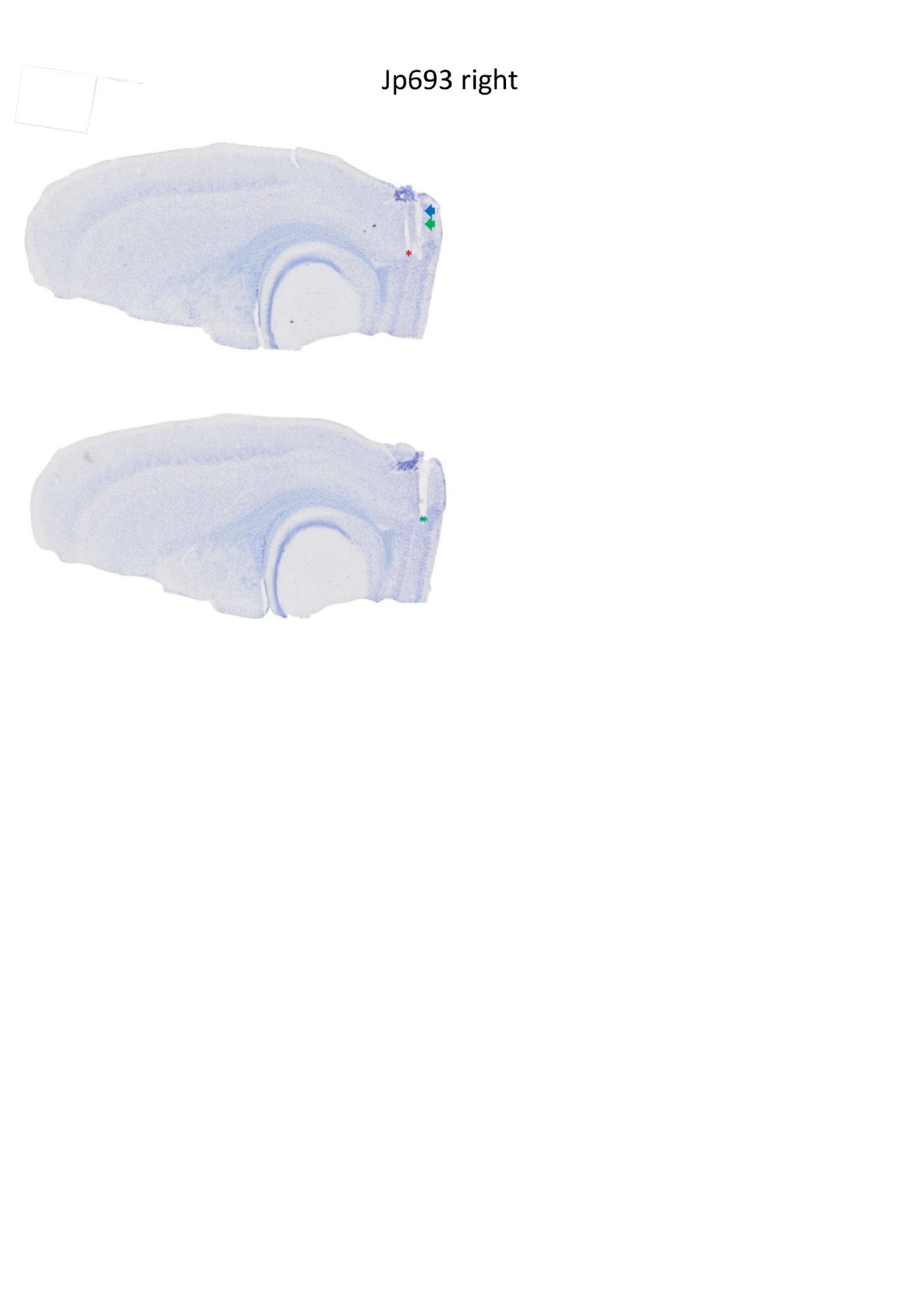

Supplement: Figure 1—source data 1. — Each page of the document shows the brain sections with tetrode tracks of one hemisphere. Arrows point to the tetrode tracks and asterisks indicate the tetrode tips. Different colors were assigned to different tetrodes. DOI: http://dx.doi.org/10.7554/eLife.16937.003 [file elife-16937-fig1-data1.pptx]

## Slide 1
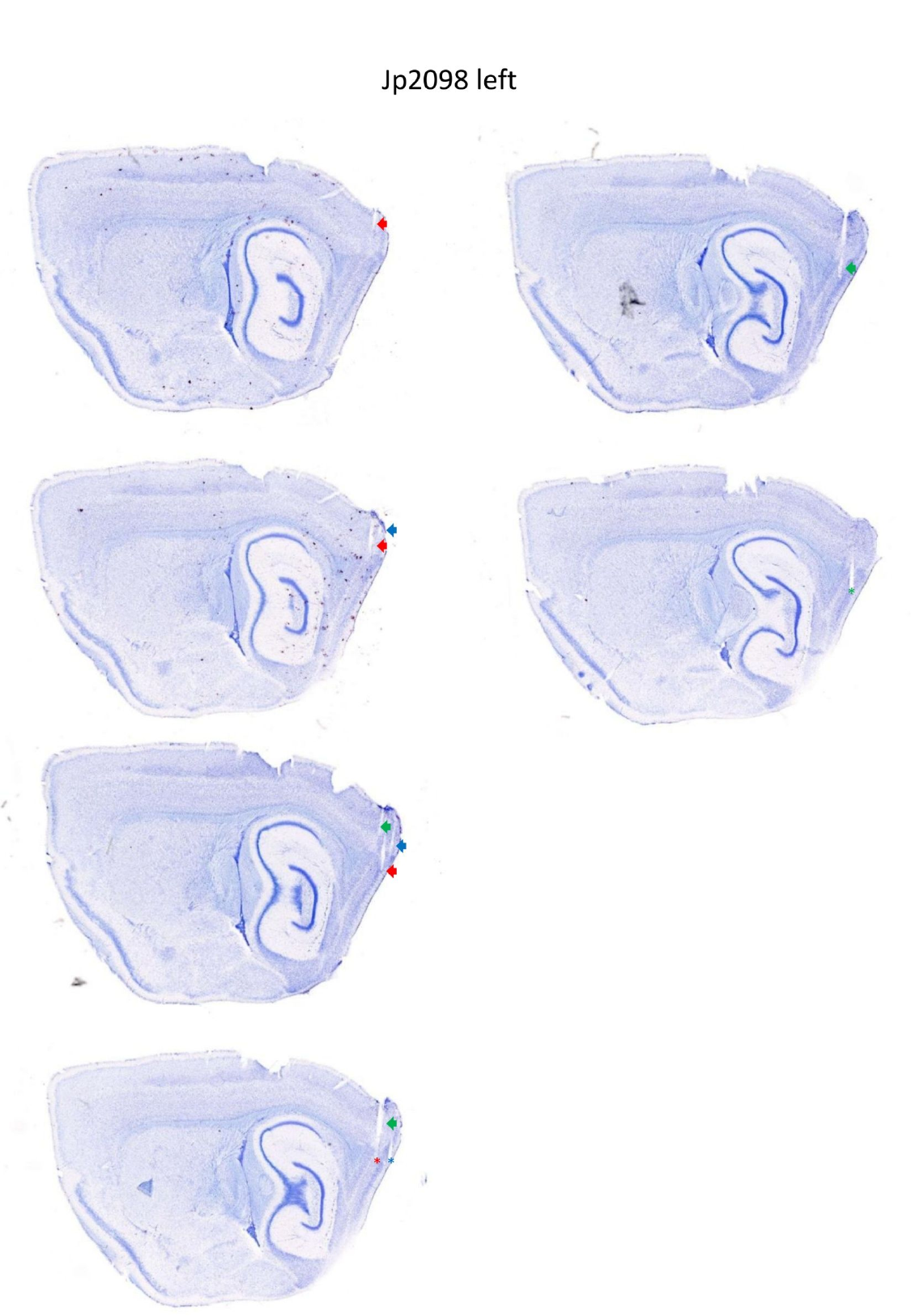

## Slide 2
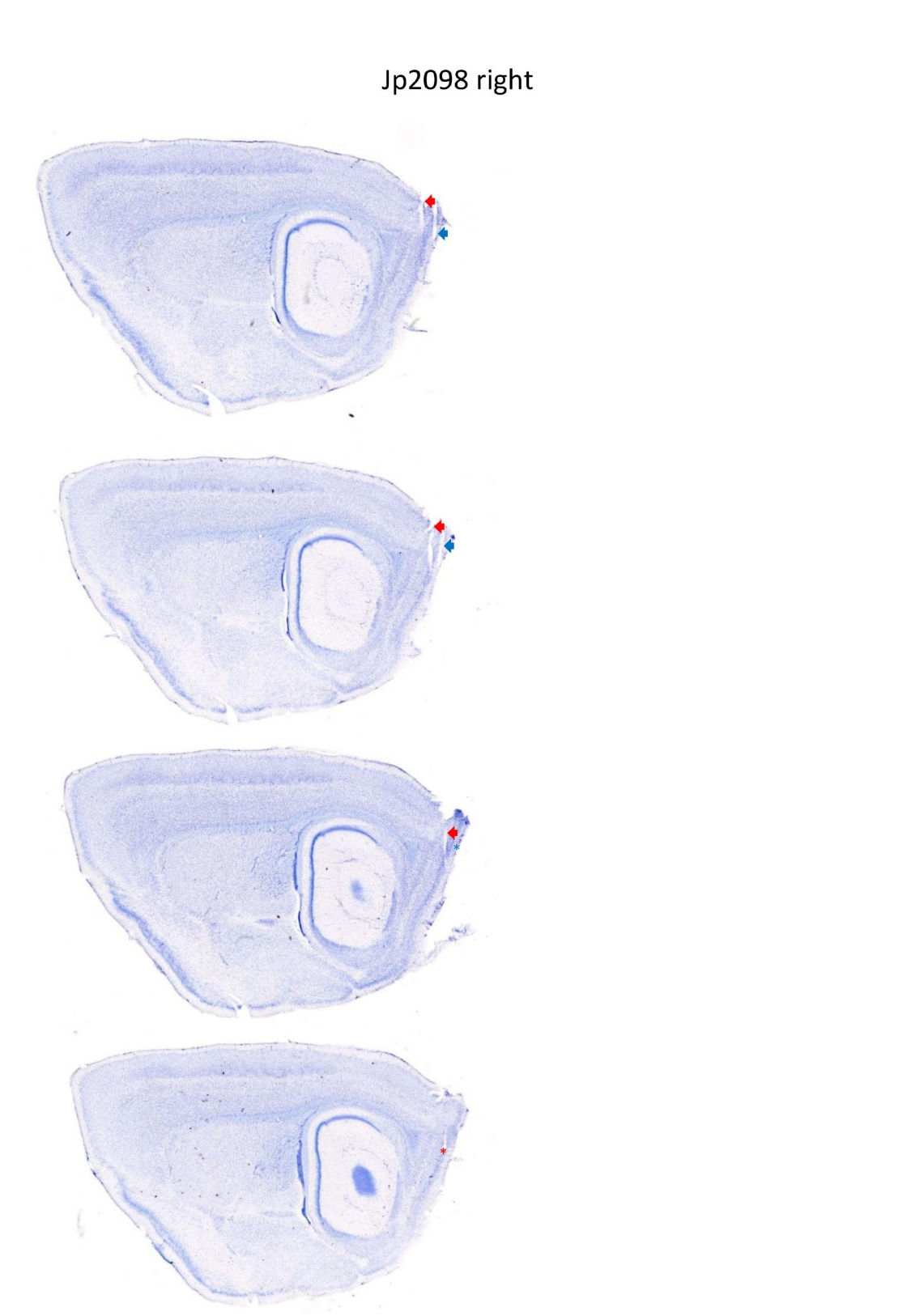

## Slide 3
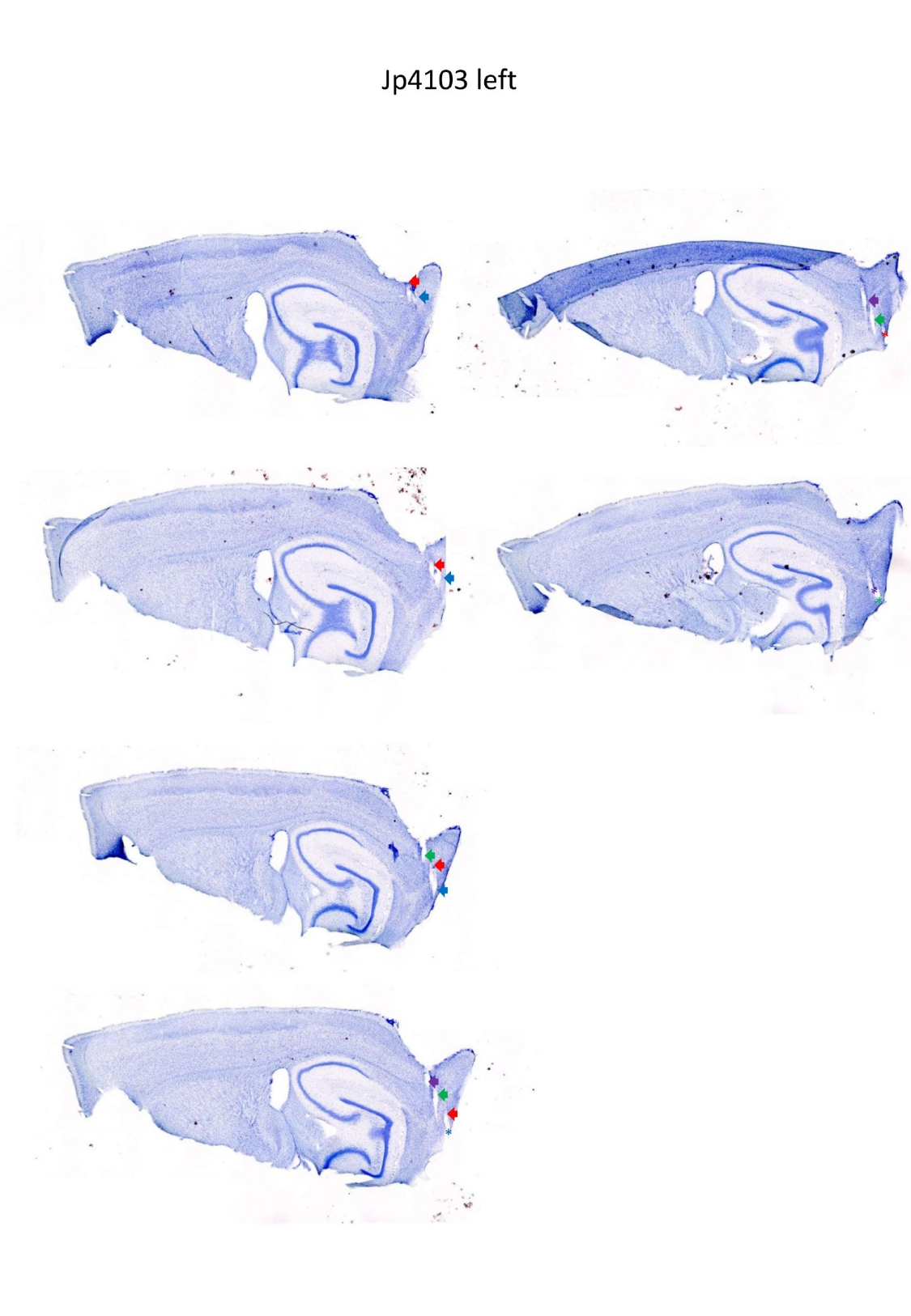

## Slide 4
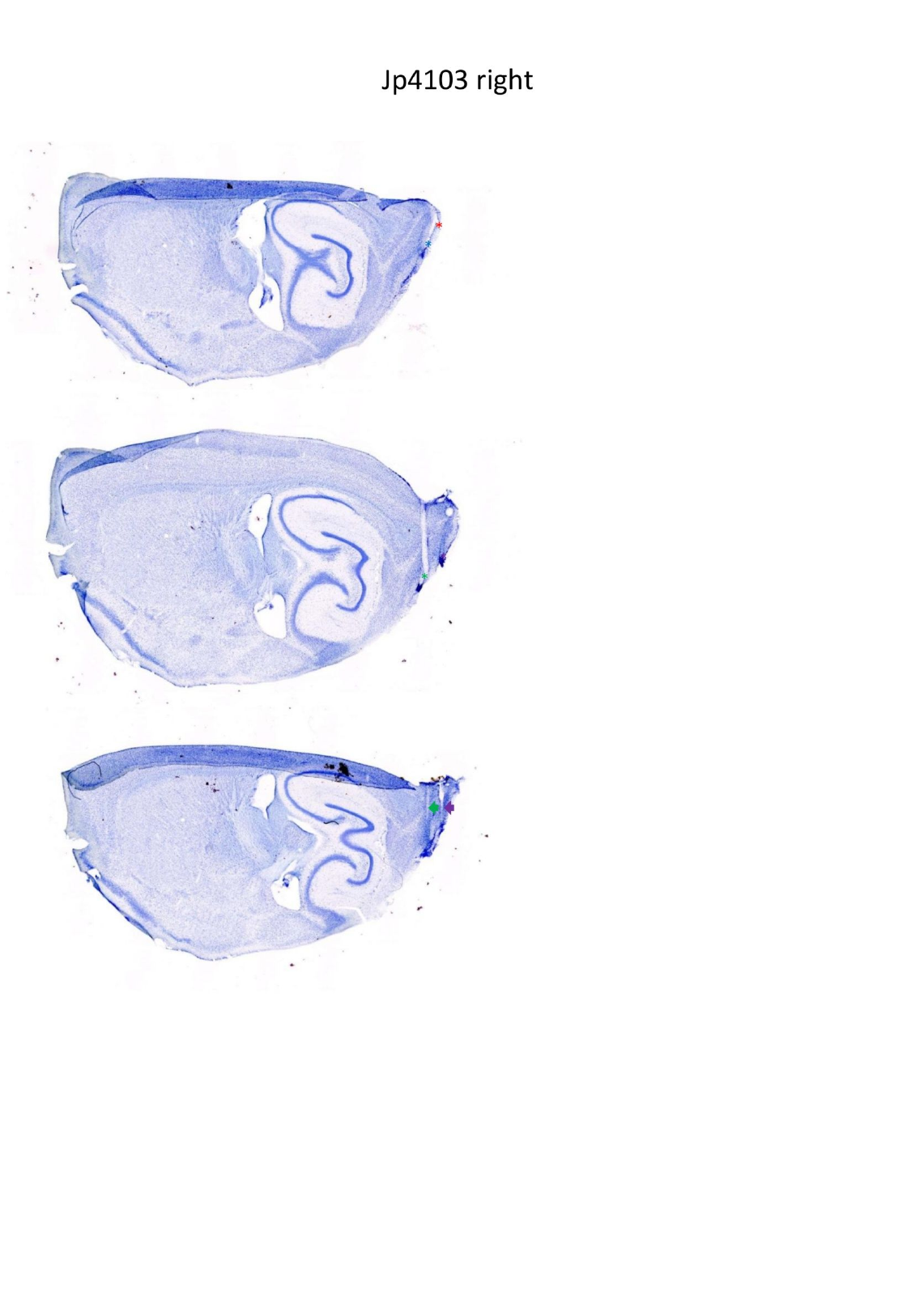

## Slide 5
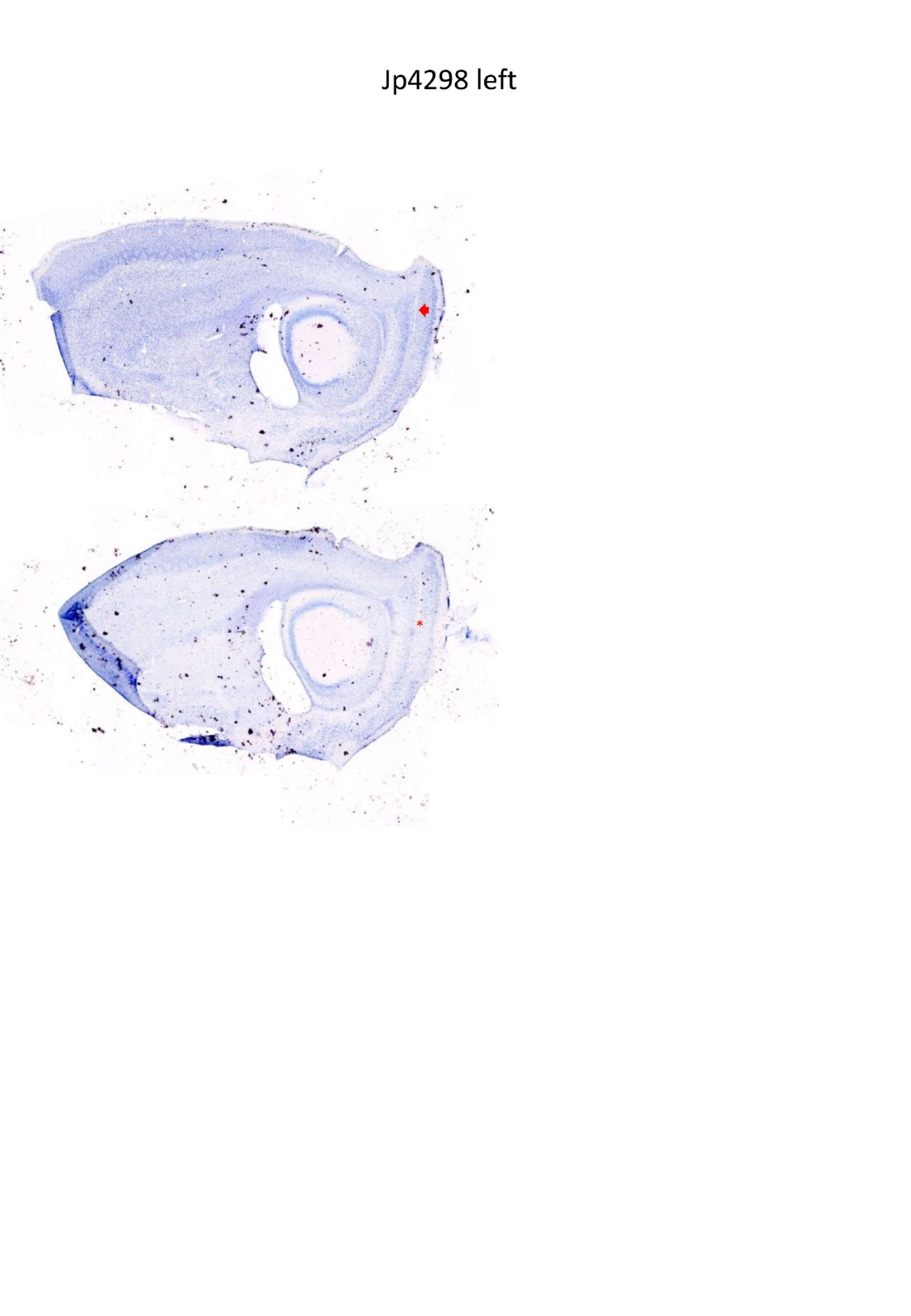

## Slide 6
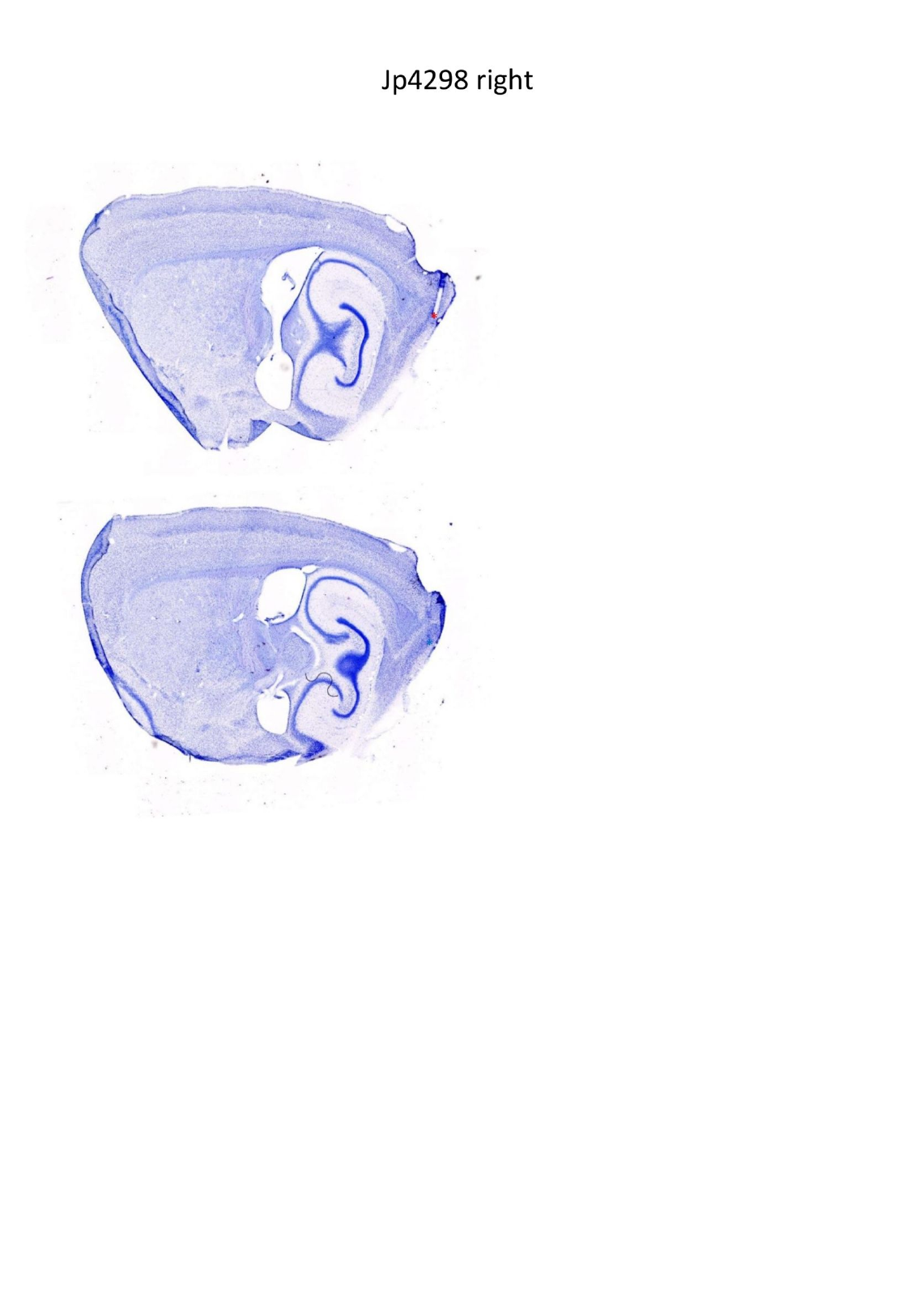

## Slide 7
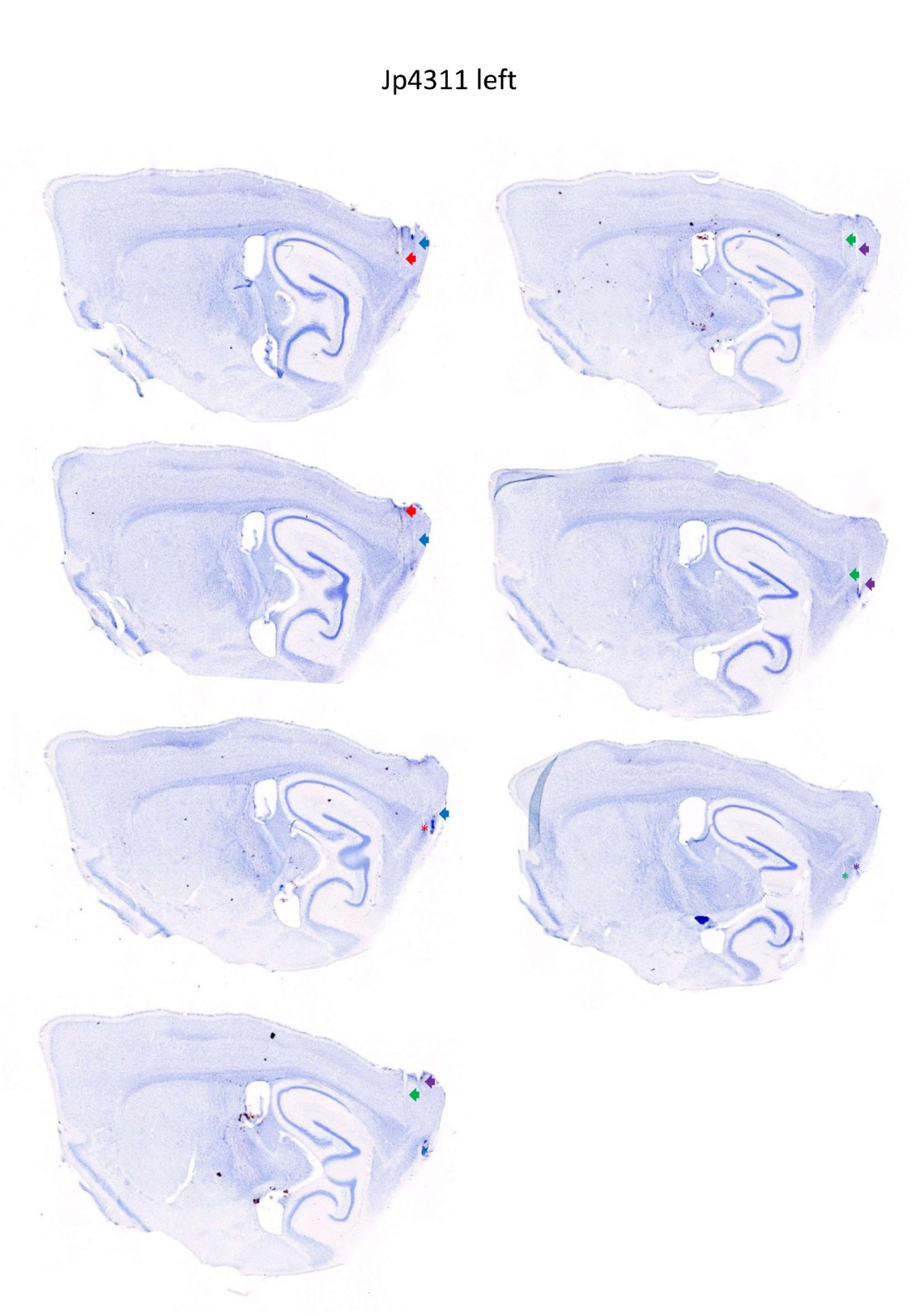

## Slide 8
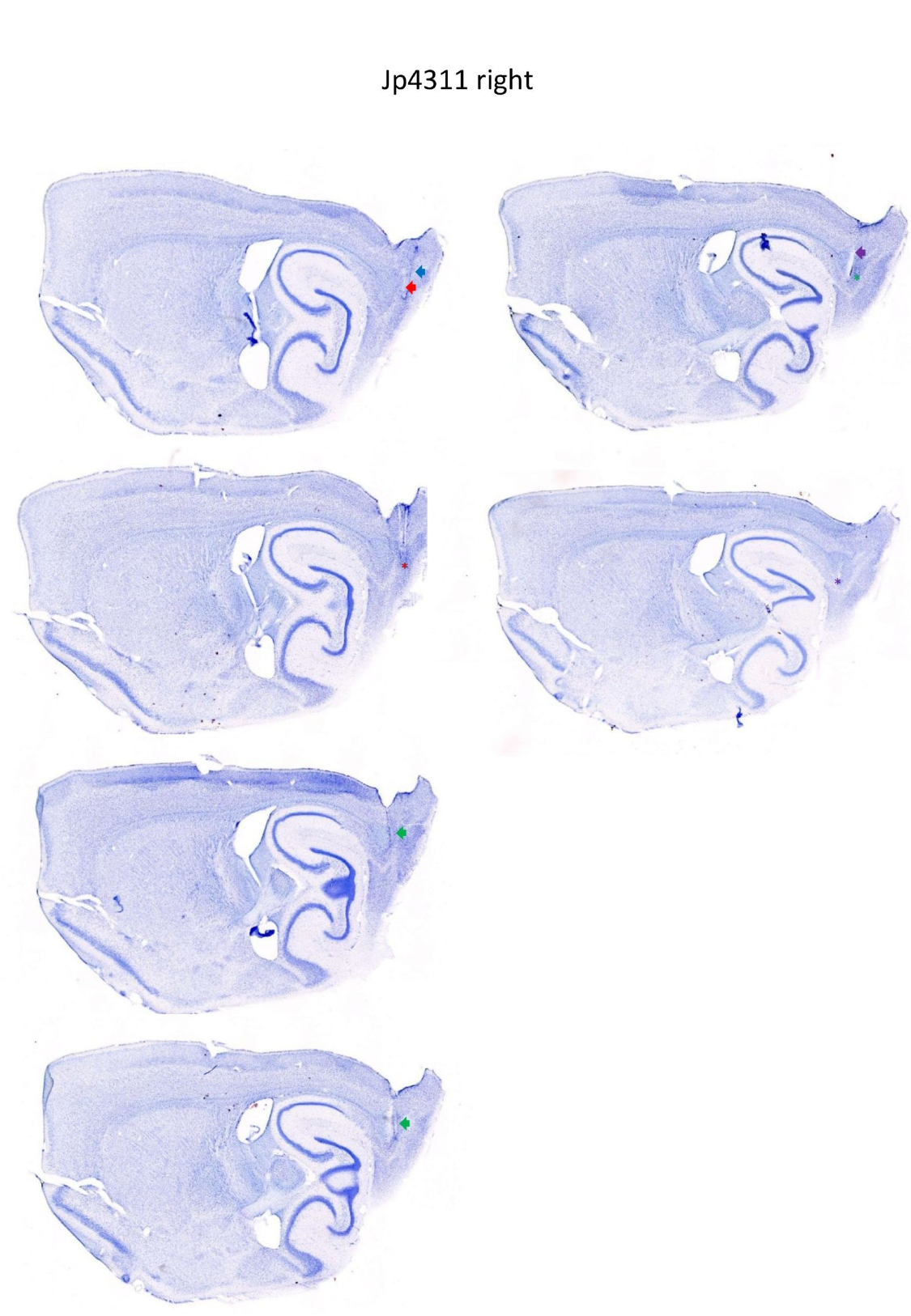

## Slide 9
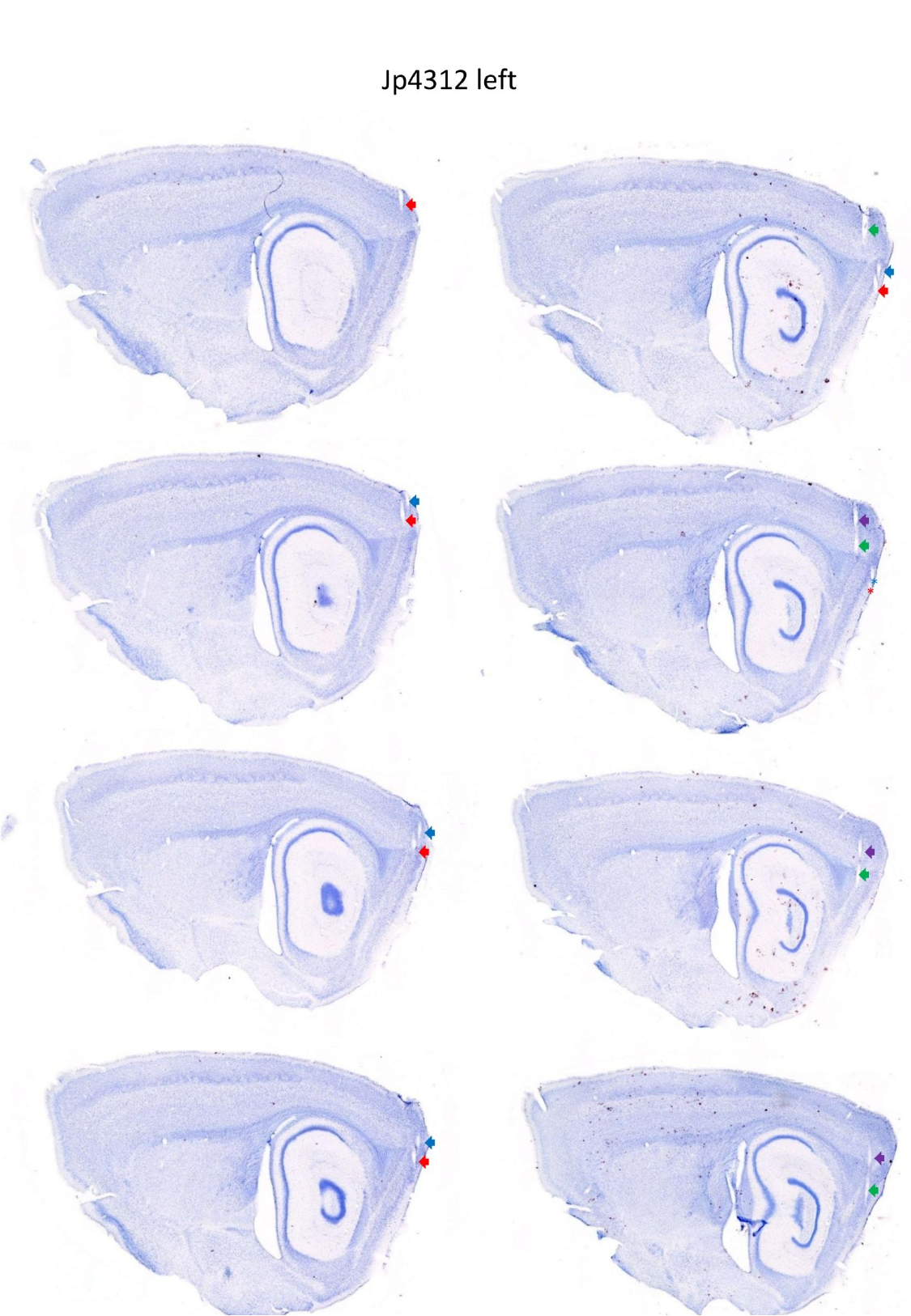

## Slide 10
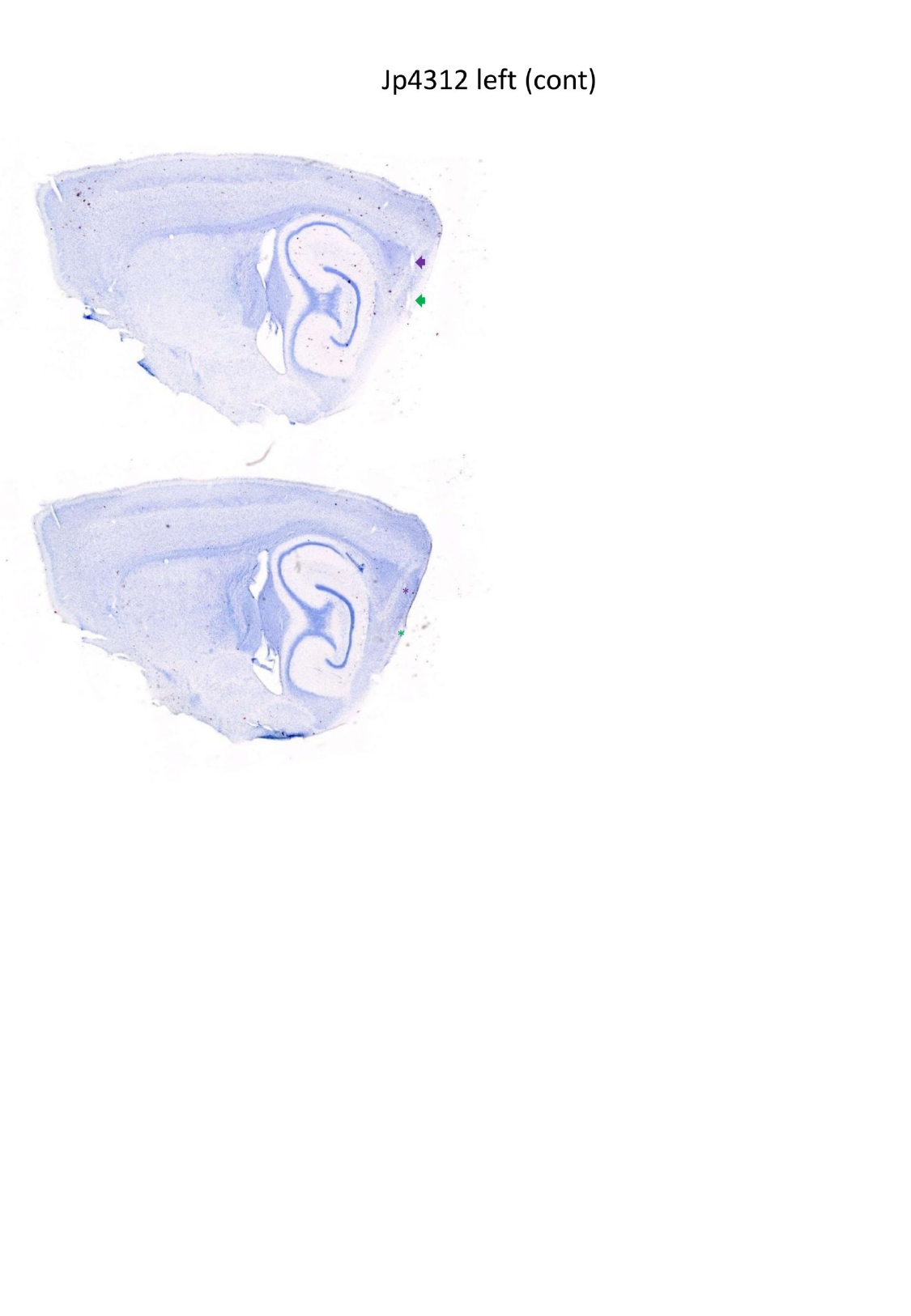

## Slide 11
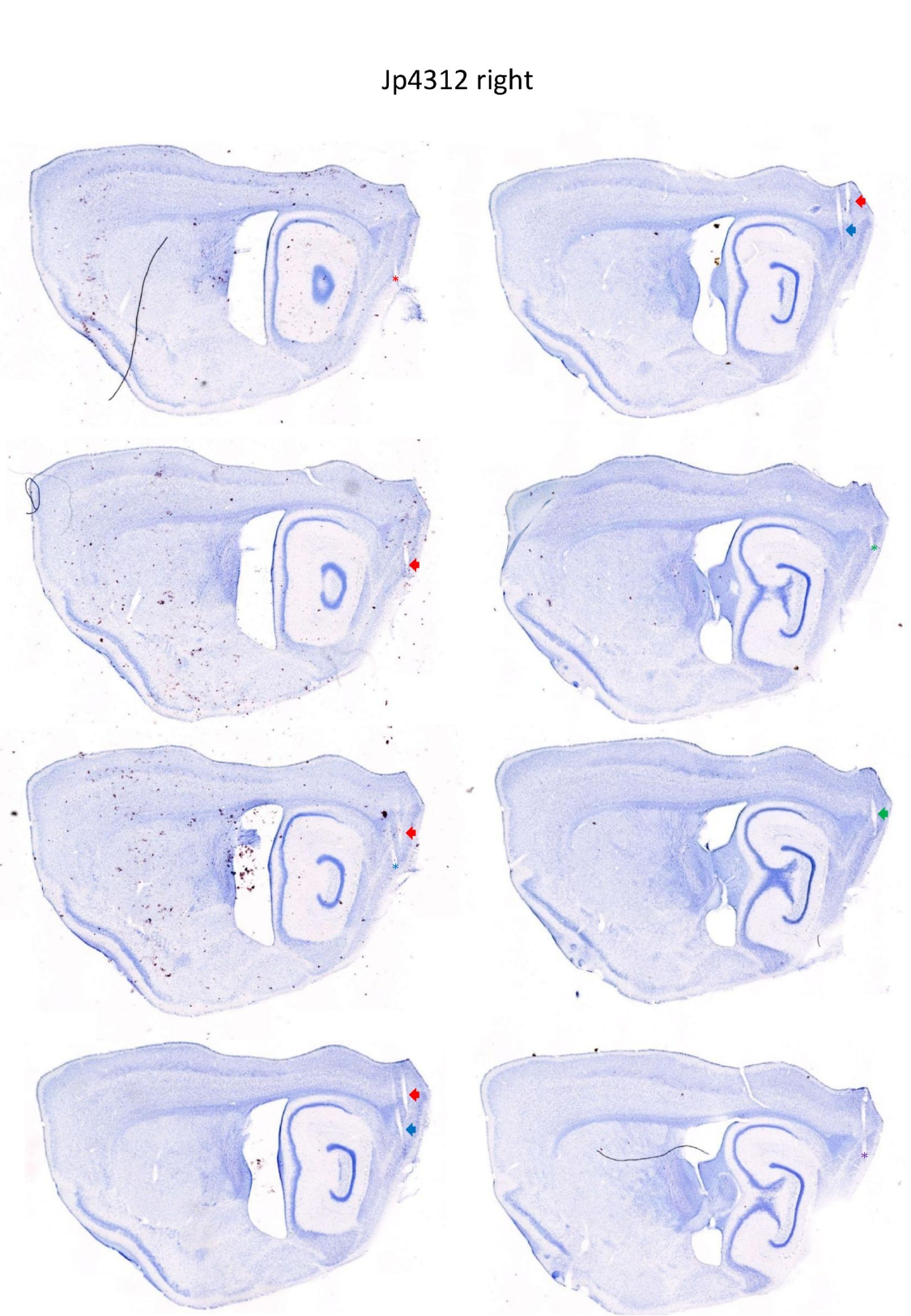

## Slide 12
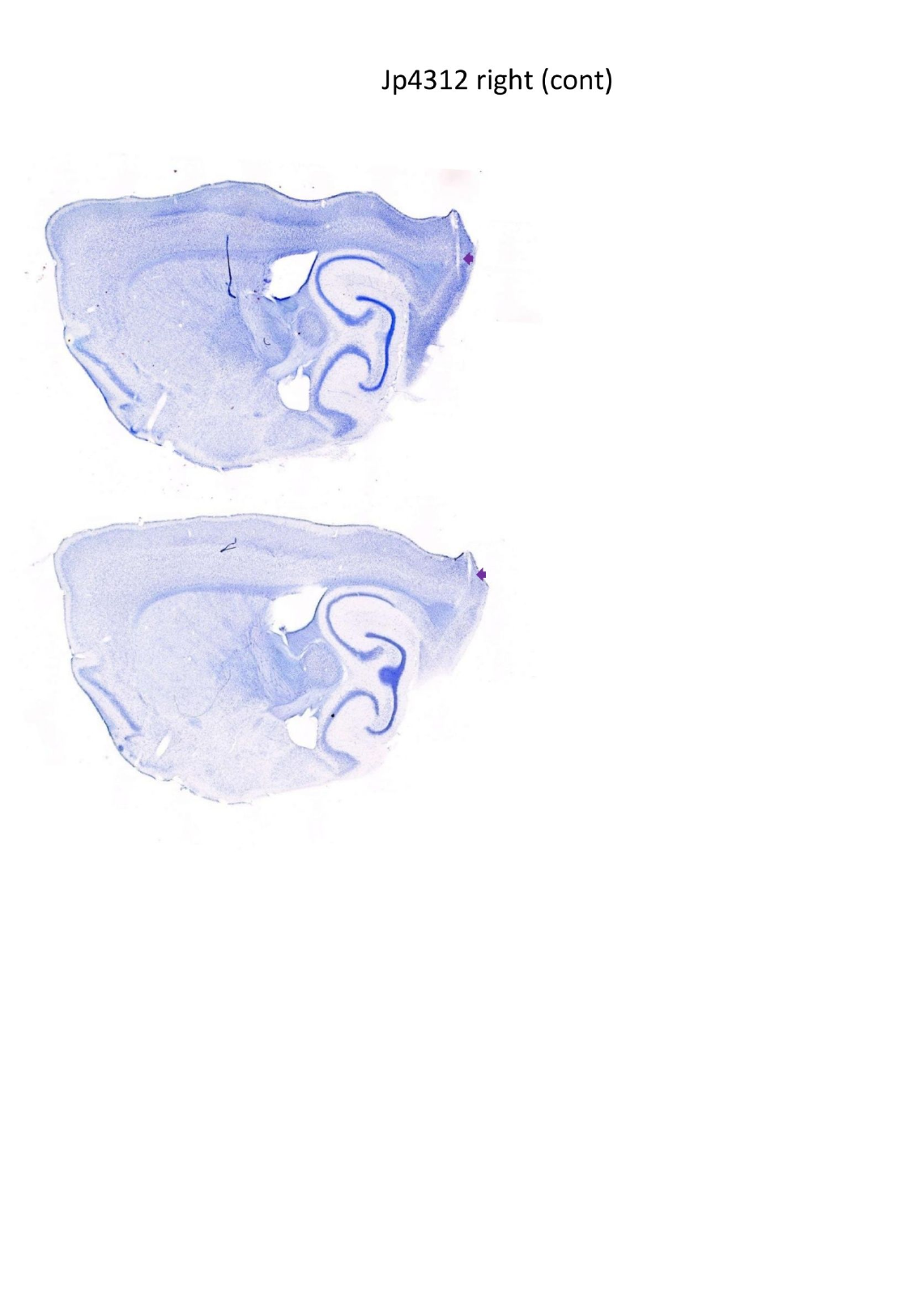

Supplement: Figure 8—source data 1. — Each page of the document shows the brain sections with tetrode tracks of one hemisphere. Arrows point to the tetrode tracks and asterisks indicate the tetrode tips. Different colors were assigned to different tetrodes. DOI: http://dx.doi.org/10.7554/eLife.16937.014 [file elife-16937-fig8-data1.pptx]
