# Supplementary material for: Visual landmarks sharpen grid cell metric and confer context specificity to neurons of the medial entorhinal cortex
Source: eLife. 2016 Jul 23;5:e16937. doi: 10.7554/eLife.16937 (PMC4987135; doi:10.7554/eLife.16937)
Supplement: Figure 1—source data 2. — * Extensive cortical damage; tetrodes from this hemisphere were not considered as MEC tetrodes in the analysis. ** Histological results unavailable. DOI: http://dx.doi.org/10.7554/eLife.16937.004 [file elife-16937-fig1-data2.docx]

| Mouse | Experiment | Hemisphere | Mec | Mec/  parasubiculum | Parasubiculum |
| --- | --- | --- | --- | --- | --- |
| Jp5519 | Circular arena | Left | 2 | 0 | 0 |
| Jp5519 | Circular arena | Right | 2 | 0 | 0 |
| Jp5520 | Circular arena | Left | 4 | 0 | 0 |
| Jp5520 | Circular arena | Right | 4 | 0 | 0 |
| Jp19843 | Circular arena | Left | 2 | 2 | 0 |
| Jp19843* | Circular arena | Right | 3 | 0 | 0 |
| Jp19844 | Circular arena | Left | 0 | 0 | 4 |
| Jp19844 | Circular arena | Right | 4 | 0 | 0 |
| Jp2142 | Circular arena | Left | 2 | 2 | 0 |
| Jp2142 | Circular arena | Right | 2 | 2 | 0 |
| Jp21414 | Circular arena | Left | 1 | 0 | 2 |
| Jp21414 | Circular arena | Right | 4 | 0 | 0 |
| Jp693 | Circular arena | Left | 4 | 0 | 0 |
| Jp693 | Circular arena | Right | 3 | 0 | 0 |
| Jp19841** | Circular arena | Left |  |  |  |
| Jp19841** | Circular arena | Right |  |  |  |
| **Total** | **Circular arena** |  | **37** | **6** | **6** |

*Extensive cortical damage

**Histology unavailable
