# Supplementary material for: Visual landmarks sharpen grid cell metric and confer context specificity to neurons of the medial entorhinal cortex
Source: eLife. 2016 Jul 23;5:e16937. doi: 10.7554/eLife.16937 (PMC4987135; doi:10.7554/eLife.16937)
Supplement: Figure 8—source data 2. — * One tetrode tip was located in the postsubiculum. DOI: http://dx.doi.org/10.7554/eLife.16937.015 [file elife-16937-fig8-data2.docx]

| Mouse | Experiment | Hemisphere | Mec | Mec/  parasubiculum | Parasubiculum |
| --- | --- | --- | --- | --- | --- |
| Jp2098 | Linear track | Left | 3 | 0 | 0 |
| Jp2098 | Linear track | Right | 2 | 0 | 0 |
| Jp4103 | Linear track | Left | 0 | 0 | 4 |
| Jp4103 | Linear track | Right | 2 | 0 | 2 |
| Jp4298 | Linear track | Left | 1 | 0 | 0 |
| Jp4298 | Linear track | Right | 1 | 0 | 1 |
| Jp4311 | Linear track | Left | 0 | 2 | 2 |
| Jp4311* | Linear track | Right | 0 | 0 | 3 |
| Jp4312 | Linear track | Left | 2 | 1 | 1 |
| Jp4312 | Linear track | Right | 3 | 1 | 0 |
| **Total** | **Linear track** |  | **14** | **4** | **13** |

*One tetrode tip was located in the postsubiculum
